# Obesity is associated with a decrease in expression but not with the hypermethylation of thermogenesis-related genes in adipose tissues

**DOI:** 10.1186/s12967-015-0395-2

**Published:** 2015-01-27

**Authors:** Alina Kurylowicz, Marta Jonas, Wojciech Lisik, Maurycy Jonas, Zofia Agnieszka Wicik, Zbigniew Wierzbicki, Andrzej Chmura, Monika Puzianowska-Kuznicka

**Affiliations:** Department of Human Epigenetics, Mossakowski Medical Research Centre, Polish Academy of Sciences, 5 Pawinskiego Street, 02-106 Warsaw, Poland; Department of General and Transplantation Surgery, Medical University of Warsaw, 59 Nowogrodzka Street, 02-005 Warsaw, Poland; Department of Geriatrics and Gerontology, Medical Centre of Postgraduate Education, 99 Marymoncka Street, 01-813 Warsaw, Poland

**Keywords:** Obesity, Adrenergic receptors, Thyroid hormone receptors, 5’-iodothyronine deiodinases, Uncoupling proteins, Methylation

## Abstract

**Background:**

Impaired thermogenesis can promote obesity. Therefore, the aim of this study was to investigate whether the expression of thermogenesis-related genes is altered in adipose tissues of obese individuals and whether excessive methylation of their promoters is involved in this phenomenon.

**Methods:**

The expression of genes encoding β adrenergic receptors (*ADRBs*), thyroid hormone receptors (*THRs*), 5’-iodothyronine deiodinases (*DIOs*), and uncoupling proteins (*UCPs*) was measured by real-time PCR in visceral and in subcutaneous adipose tissues of 58 obese (BMI >40 kg/m^2^) and 50 slim (BMI 20-24.9 kg/m^2^) individuals. The methylation status of these genes was studied by the methylation-sensitive digestion/real-time PCR method.

**Results:**

The expression of *ADRB2*, *ADRB3*, *THRA*, *THRB*, *DIO2, UCP2* was significantly lower in the adipose tissues of obese patients than in tissues of normal-weight individuals (P < 0.00001). In the obese, the expression of *ADRB2*, *ADRB3*, *DIO2* was lower in visceral adipose tissue than in subcutaneous adipose tissue (P = 0.008, P = 0.002, P = 0.001, respectively). However, the mean methylation of CpG islands of these genes was similar in tissues with their high and low expression, and there was no correlation between the level of expression and the level of methylation.

**Conclusions:**

Decreased expression of thermogenesis-related genes in adipose tissues of obese patients might result in the reduced reactivity to both hormonal and adrenergic stimuli and therefore in a lower potential to activate thermogenesis.

**Electronic supplementary material:**

The online version of this article (doi:10.1186/s12967-015-0395-2) contains supplementary material, which is available to authorized users.

## Background

The imbalance between energy intake and expenditure is one of the main causes of obesity [1]. Since most of the currently available non-invasive obesity treatments are ineffective in the long-term, new therapeutic strategies need to be developed to reduce excessive adiposity [2].

In small mammals and in human newborns, non-shivering thermogenesis in brown adipose tissue (BAT) is the most important regulatory mechanism for maintaining body temperature [3]. Functional studies proved however that BAT cells dispersed within the white adipose tissue (WAT), also known as beige/brite adipocytes, co-regulate energy homeostasis and their number increases upon exposure to cold also in adult organisms [4,5]. Thermogenic function of brown adipocytes is under strict neurohormonal control. Exposure to cold leads to increased noradrenaline release and subsequent stimulation of various subtypes of β-adrenergic receptors (ADRB), leading to the proliferation of brown adipocytes (ADRB1) [6] and activation of lipolysis and/or of thermogenesis (ADRB2 and ADRB3) [7]. While adrenergic stimulation provides the means for rapid responses, triiodothyronine (T3) increases the capacity of cells to respond to catecholamines [8,9]. Moreover, cold-induced noradrenergic stimulation of brown adipocytes results in the local activation of the type 2 5’-iodothyronine deiodinase (DIO2), which catalyzes conversion of thyroxin (T4) to T3 [10]. Subsequently, T3 acting by its α and β nuclear receptors (TRα and TRβ, respectively) increases the expression of various genes, including the genes for uncoupling proteins (UCP) responsible for dissipation of oxidation energy as heat in BAT’s mitochondria [11,12].

There is mounting evidence that disturbances of thermogenesis-related pathways may play a role in the development of obesity. Early epidemiological studies showed that obese individuals have lower energy expenditure; this could be explained by a lower efficiency of adaptive thermogenesis [13]. To verify this hypothesis, a number of animal knock-outs with a selective ablation of various thermogenesis-related genes were created; however, results of these studies were ambiguous and underlined the complexity of mechanisms controlling thermogenesis [14-18]. Studies concerning the use of various activators of thermogenesis in the treatment of obesity were also performed, and a number of novel thermogenic compounds are currently being studied, such as synthetic and selective thyroid hormone receptor or β-adrenergic receptor agonists [19].

In recent years, epigenetic modifications, activated *via* environmental stimuli, has been found to be an important mechanism regulating the gene expression in adipocytes. For example, it was shown that in mouse pre-adipocytes, a high-fat diet promotes methylation of the peroxisome proliferator-activated receptor γ (*PPARG*) promoter and this may contribute to the pathogenesis of obesity and of the metabolic syndrome [20]. Diet deficient in methyl group donors and methionine-homocysteine cycle co-factors (e.g. folate, methionine, choline, betaine) reduces methylation and up-regulates the expression of genes involved in free fatty acids uptake and in synthesis of triglycerides, promoting fatty-liver disease and, presumably, obesity [21]. In human adipose tissue, even moderate calorie restriction decreases methylation of the cholesterol ester transfer protein gene resulting in its higher expression; this facilitates lipid transport to cellular sites where hydrolytic enzymes are active [22]. Therefore, it is plausible that epigenetic modifications regulate the equilibrium between the environment and the activity of thermogenesis-related genes. This is supported by the results of animal studies showing that the expression of these genes depends on environmental factors such as diet or exposure to cold (14-17).

So far little is known about the physiological changes in thermogenesis-related pathways in adipose tissue of obese humans, and such knowledge would constitute a crucial link between the *in vitro* experiments and pharmacological studies. In this work we show that the expression of several thermogenesis-related genes is lower in adipose tissues originating from obese individuals than in tissues of non-obese study participants, and that the level of expression of these genes is probably not related to the methylation status of their promoters.

## Methods

### Adipose tissue

One hundred sixteen samples of visceral (VAT) and subcutaneous (SAT) adipose tissues were obtained from 58 obese patients (body mass index (BMI) calculated as weight (kg) divided by height squared (m^2^), >40 kg/m^2^) during bariatric surgery. Fifty control tissues were collected from normal-weight patients (BMI 20-24.9 kg/m^2^) undergoing elective cholecystectomy (VAT, N = 22) or operated on for inguinal hernia (SAT N = 28). After collection, the samples were immediately frozen in liquid nitrogen and stored at -80°C. The project was approved by the Bioethics Committee of the Medical University of Warsaw (decision KB/47/2009), and a written informed consent for participation in this study was obtained from all participants.

### Basic biochemical parameters

Basic biochemical and hormonal parameters in serum/plasma of obese individuals were measured in the diagnostic laboratory of the Infant Jesus Teaching Hospital of the Medical University of Warsaw, according to a routine procedure.

### Nucleic acids isolation, reverse transcription and real-time PCR

Approximately 500 mg of each tissue was homogenized in liquid nitrogen and total RNA and DNA were extracted with TRIzol Reagent (Invitrogen, Carlsbad, CA, USA) according to the manufacturer’s procedure. One hundred nanograms of each RNA was used for reverse transcription performed with RevertAid First Strand cDNA Synthesis Kit (Fermentas, Vilnius, Lituania) according to the manufacturer’s protocol. The resulting cDNA was diluted in RNAse-free dH_2_O (Invitrogen, Carlsbad, CA, USA). Next, 1 μl of cDNA corresponding to 0.5 ng of total RNA was used as a template in real-time PCR performed in LightCycler 480 Instrument II (Roche, Mannheim, Germany) with LightCycler 480 Sybr Green I Master Kit (Roche, Mannheim, Germany) and with specific primers (Additional file 1: Table S1). The PCR conditions were as follows: initial incubation at 95°C for 10 min, 40 cycles of 95°C for 12 s, 58-62°C for 12 s, 72°C for 12 s, and then one melting curve cycle. All measurements were performed in triplicate. The results were normalized against the results for the β-actin gene (*ACTB*) and presented in arbitrary units (AU) as mean mRNA levels, as well as the mean expression Fold Change (FC = 2^-ΔΔCt^), considering that FC is significant if < -1.50 (down-regulation) or >1.50 (up-regulation).

### Methylation analysis

Analysis of methylation was performed with the OneStep qMethyl Kit (Zymo Research, Irvine, CA, USA) according to the manufacturer’s protocol. The tested DNA was divided into a “test reaction” and a “reference reaction”. Each sample contained 20 ng of the genomic DNA, 10 μl of 2x Test/Reference Reaction Premix and 1 μl of 10 μM solution of the primers (Additional file 2: Table S2). The test sample was then digested with a mix of methylation sensitive restriction enzymes (*Acc*II, *Hpa*II, *HpyCHIV*4) at 37°C for 2 hours, while the reference sample was not. Subsequently, the DNA from both samples was amplified in the LightCycler 480 Instrument II in the presence of SYTO® 9 fluorescent dye and then quantified. The PCR conditions were as follows: denaturation at 95°C for 10 min, 45 cycles of 95°C for 30 s, 62-64°C for 60 s, 72°C for 60 s, and then one melting curve cycle. All measurements were performed in duplicate. Along with the patients’ samples, Human Methylated & Non-methylated DNA Standards provided by the manufacturer were also tested in order to validate the quality of the reactions. The methylation level of the amplified region was determined using the following equation: percent methylation = 100 × 2^-ΔCt^, where ΔCt is the average Ct value from the test reaction minus the average Ct value from the reference reaction.

### Statistical analysis

The differences in mRNA expression were assessed with the Statistica software package v.10 (StatSoft, Tulsa, OK) using the Student’s t/Mann-Whitney U test or Kruskal-Wallis analysis of variance. All correlations between quantitative values were performed with the Spearman correlation test. Normality of distribution and homogeneity of the variance were checked with the Shapiro-Wilk and Levene’s tests, respectively. To minimize false positives, the Bonferroni correction for multiple testing was applied and the level of significance was established at 0.01.

## Results

### Studied groups

The group of obese individuals consisted of 49 females and 9 males. Their basic clinical characteristics, biochemical parameters and thyroid status are summarized in Table 1. The metabolic syndrome was diagnosed in 48 (82.7%) on the basis of the International Diabetes Federation criteria for the Europeans [www.idf.org]. All patients suffering from type 2 diabetes mellitus and with a glomerular filtration rate (GFR) ≥60 ml/min were treated with metformin or with a combination of metformin and sulphonylureas, while individuals with GFR <60 ml/min were treated only with sulphonylureas. Patients with hypertension received at least one antihypertensive drug (angiotensin-converting enzyme inhibitor, angiotensin II receptor antagonist, diuretic, calcium channel blocker, or β-blocker). Individuals diagnosed with hyperlipidemia received statins or fibrates. All patients were euthyroid.

**Table 1:** **Selected clinical and biochemical parameters of study participants**

	**Obese individuals**		**Controls**	
	**Mean ± SD**	**Min-Max**	**Mean ± SD**	**Min-Max**
Age (years)	41.45 (±10.18)	20-59	44 (±14.1)	23-62
Weight (kg)	131.98 (±17.72)	100-199.8	67 (±11.23)	(50-90)
BMI (kg/m^2^)	46.93 (±4.77)	40.11-59.25	23.32 (±1.57)	20.1-24.9
Adipose tissue (% body mass)	48.08 (±4.95)	32.64-57.23	-	
Waist circumference (cm)	123.24 (±18.34)	97-167	-	
CRP (nmol/l)	95.6 (±44.1)	11.4-184.7	26.19 (±18.1)	(1.9-49.5)
Glucose (mmol/l)	5.57 (±1.47)	3.22-10.16	4.99 (±0.41)	(4.22-5.49)
Total cholesterol (mmol/l)	5.21 (±1.54)	3.13-7.86	5.04 (±0.23)	(4.81-5.33)
LDL (mmol/l)	3.26 (±1.03)	1.24-5.64	2.74 (±0.13)	(2.64-2.9)
HDL (mmol/l)	1.22 (±0.24)	0.77-1.79	1.63 (±0.39)	(1.24-2.15)
Triglycerides (mmol/l)	1.70 (±1.36)	0.52-7.62	1.29 (±0.17)	(1.09-1.46)
TSH (IU/l)	1.70 (±0.85)	0.33-3.65	1.22 (±0.18)	1,09-1,35
FT4 (pmol/l)	13.82 (±3.13)	8.4-23.15	-	
FT3 (nmol/l)	2.85 (±1.01)	2.06-3.05	-	
Co-morbidities				
Type 2 DM/IGT	26 (44.83%)	none		
Hypertension	35 (60.34%)	none		
Hyperlipidemia	36 (62.09%)	none		

BMI: body mass index calculated as weight (kg) divided by height squared (m^2^), LDL: low density lipoproteins, HDL: high density lipoproteins, CRP: C-reactive protein, TSH: thyroid stimulating hormone, FT4: free thyroxin, FT3: free triiodothyronine, DM: diabetes mellitus, IGT: impaired glucose tolerance.

The control group consisted of 40 females and 10 males. Apart from cholelithiasis or inguinal hernia, they had no history of any chronic disease, including components of the metabolic syndrome and thyroid diseases. Their normal health status was confirmed by physical examination and blood tests (Table 1). Although their adipose tissue content was not calculated, based on their medical history, BMI values and biochemical parameters, they were considered to be metabolically healthy.

A detailed dietary questionnaire was not obtained from our study subjects; however, calculations based on the typical menus showed that in obese individuals the average daily calorie intake exceeded energy demand by at least 1000-1500 kcal in women and by 1500-2000 kcal in men. All obese individuals led a sedentary lifestyle.

### Expression of thermogenesis-related genes in adipose tissues from obese and from slim individuals

Ten genes were included into the study: *ADRB1*, *ADRB2* and *ADRB3* encoding adrenergic receptors β1, β2 and β3, *DIO1* and *DIO2* encoding type 1 and type 2 5’-iodothyronine deiodinases, *THRA* and *THRB* encoding thyroid hormone receptors α and β, and *UCP1*, *UCP2*, *UCP3* encoding uncoupling proteins 1, 2 and 3. Initial analysis showed that the mean expressions of these genes did not differ in adipose tissues of males and females; therefore, all analyses were performed for both sexes together.

**Figure 1:**
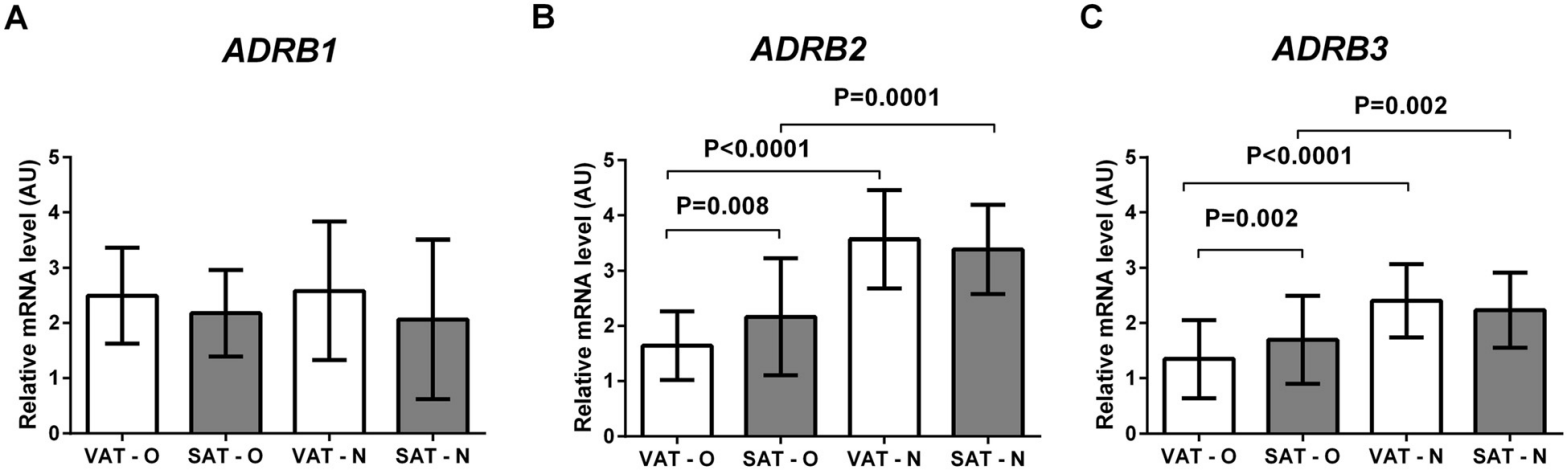
**Comparison of the expression of** ***ADRB1*** **(A),** ***ADRB2*** **(B) and** ***ADRB3*** **(C) genes in the visceral (VAT) and subcutaneous (SAT) adipose tissues of obese (O) and normal-weight (N) individuals.** Results, normalized against the expression of *ACTB*, are shown as the mean ± standard deviation.

Analysis of the expression of *ADRB2* (Figure 1B) and *ADRB3* (Figure 1C) showed that their mean mRNA levels were significantly lower in adipose tissues of obese patients than in the tissues of normal-weight controls (P < 0.00001 and P < 0.00001), both in VAT (P < 0.0001 for both) and in SAT (P = 0.0001 and P = 0.002, respectively). Furthermore, in obese patients the mean expression of *ADRB2* and *ADRB3* was significantly lower in VAT than in SAT (P = 0.008 and P = 0.002, respectively), while in normal-weight individuals no difference between VAT and SAT was detected. The expression of *ADRB1* was similar in all examined tissues (Figure 1A).

**Figure 2:**
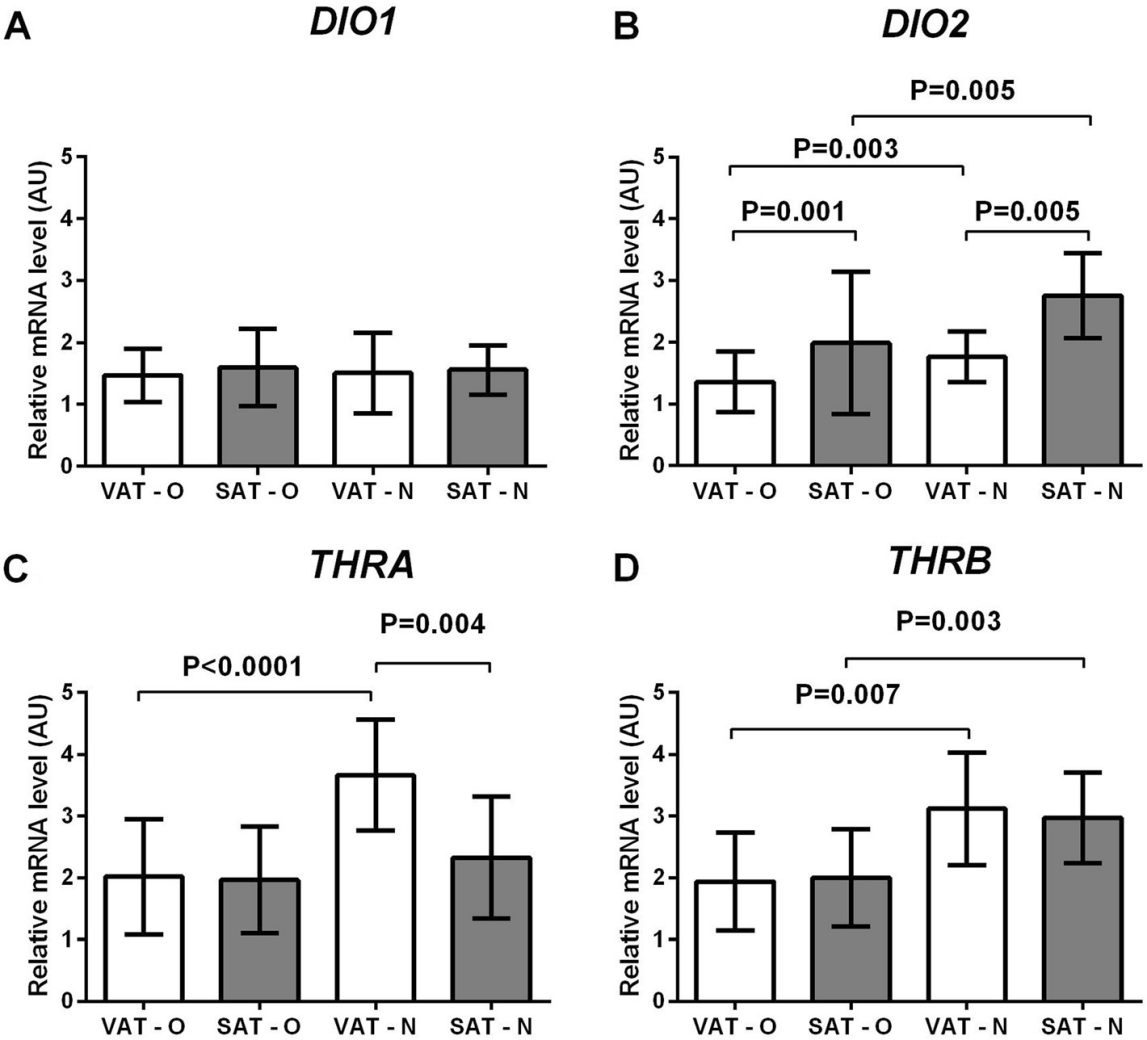
**Comparison of the expression of** ***DIO1*** **(A),** ***DIO2*** **(B),** ***THRA*** **(C), and** ***THRB*** **(D) genes in the visceral (VAT) and subcutaneous (SAT) adipose tissues of obese (O) and normal-weight (N) individuals.** Results, normalized against the expression of *ACTB*, are shown as the mean ± standard deviation.

The mean expression of *DIO2* (Figure 2B) was significantly lower in adipose tissues of obese patients than in the control group (P < 0.0001), and the difference concerned both VAT (P = 0.003) and SAT (P = 0.005). In obese patients, the mean *DIO2* expression was significantly lower in VAT than in SAT (P = 0.001) and a similar observation was made in normal-weight individuals (P = 0.005). No differences in the *DIO1* expression were observed in the investigated tissues (Figure 2A). The mean expression of *THRA* (Figure 2C) was significantly lower in adipose tissues of obese patients than in those of normal-weight individuals (P < 0.0001), but the difference was detected only in VAT (P < 0.0001). In addition, in obese patients the expression of *THRA* was similar in VAT and SAT, while in normal-weight individuals it was higher in VAT than in SAT (P = 0.004). The mean expression of *THRB* (Figure 2D) was significantly lower in adipose tissues of obese patients than of normal-weight individuals (P < 0.0001), and the difference concerned both VAT (P = 0.007) and SAT (P = 0.003). The *THRB* expression was similar in VAT and SAT of obese patients. It was also similar in both adipose tissue subtypes of normal-weight individuals.

**Figure 3:**
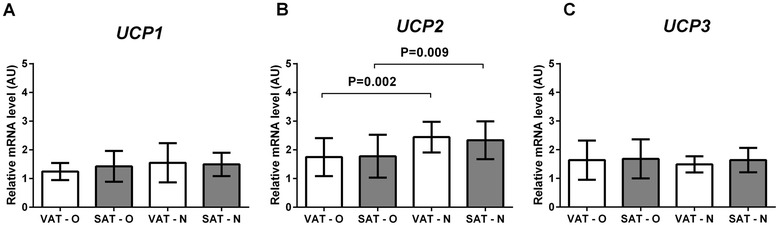
**Comparison of the expression of** ***UCP1*** **(A),** ***UCP2*** **(B), and of the** ***UCP3*** **(C) genes in the visceral (VAT) and subcutaneous (SAT) adipose tissues of obese (O) and normal-weight (N) individuals.** Results, normalized against the expression of *ACTB*, are shown as the mean ± standard deviation.

The mean expression of *UCP2* (Figure 3B) was significantly lower in adipose tissues of obese patients than in normal-weight individuals (P < 0.0001), and the difference was noted in both VAT (P = 0.002) and SAT (P = 0.009). The *UCP2* expression did not differ between VAT and SAT of obese patients and between VAT and SAT of normal-weight individuals. The analysis of *UCP1* and *UCP3* showed no significant differences in their mean mRNA levels between the investigated tissues (Figures 3A, 3C).

Alternative analysis using the expression FC (Table 2) confirmed the described above differences described above in the *ADRB2*, *ADRB3*, *DIO2*, *THRA*, *THRB* and *UCP2* mRNA levels.

**Table 2:** **Mean expression fold changes of thermogenesis-related genes in adipose tissues of obese and normal-weight individuals**

**Gene name**	**Gene description**	**Gene Bank**	**Mean expression fold change**				P*
			**VAT-O**	**SAT-O**	**VAT-N**	**SAT-N**	
*ADRB1*	adrenergic receptor beta 1	NM_000684	1.48	1.44	1.49	1.42	NS
*ADRB2*	adrenergic receptor beta 2	NM_000024	0.97	1.70	2.10	3.81	P < 0.0001
*ADRB3*	adrenergic receptor beta 3	NM_000025	2.62	1.66	5.43	3.15	P < 0.0001
*DIO1*	5’-iodothyronine deiodinase type 1	NM_000792	0.7	0.87	1.38	1.40	NS
*DIO2*	5’-iodothyronine deiodinase type 2	NM_001007023	0.55	1.62	2.13	2.60	P < 0.0001
*THRA*	thyroid hormone receptor α	NM_003250	1.79	1.70	6.03	4.18	P < 0.0001
*THRB*	thyroid hormone receptors β	NM_000461	0.57	0.58	1.92	1.13	P < 0.0001
*UCP1*	uncoupling protein 1	NM_021833	1.13	1.27	1.43	1.49	NS
*UCP2*	uncoupling protein 2	NM_003355	1.79	1.30	5.10	3.64	P < 0.0001
*UCP3*	uncoupling protein 3	NM_003356	0.82	0.53	0.85	0.86	NS

*P values calculated with the Kruskal-Wallis analysis of variance.

N: normal-weight; O: obese; VAT: visceral adipose tissue; SAT subcutaneous adipose tissue; NS: not significant.

The experiments were performed on the whole adipose tissue homogenates. However, the finding that the mean expression of two BAT-specific genes, *UCP1* and *PPARG* (Figure 3 and Additional file 3: Figure S1), was similar in all analyzed tissues, implies that the differences in the expression of thermogenesis-related genes were not due to a different content of beige adipocytes.

### Correlation between the expression of thermogenesis-related genes from adipose tissues of obese individuals and clinical, biochemical as well as hormonal parameters

The expression levels of the investigated genes were correlated with basic clinical (BMI, percentage of the adipose tissue, waist circumference), biochemical (fasting serum glucose, total cholesterol, low density lipoproteins, high density lipoproteins, triglycerides and C-reactive protein concentrations), as well as hormonal (thyroid stimulating hormone, free thyroxin, free triiodothyronine) parameters. No significant correlations were found.

### Methylation of the regulatory regions of thermogenesis-related genes in adipose tissues

**Figure 4:**
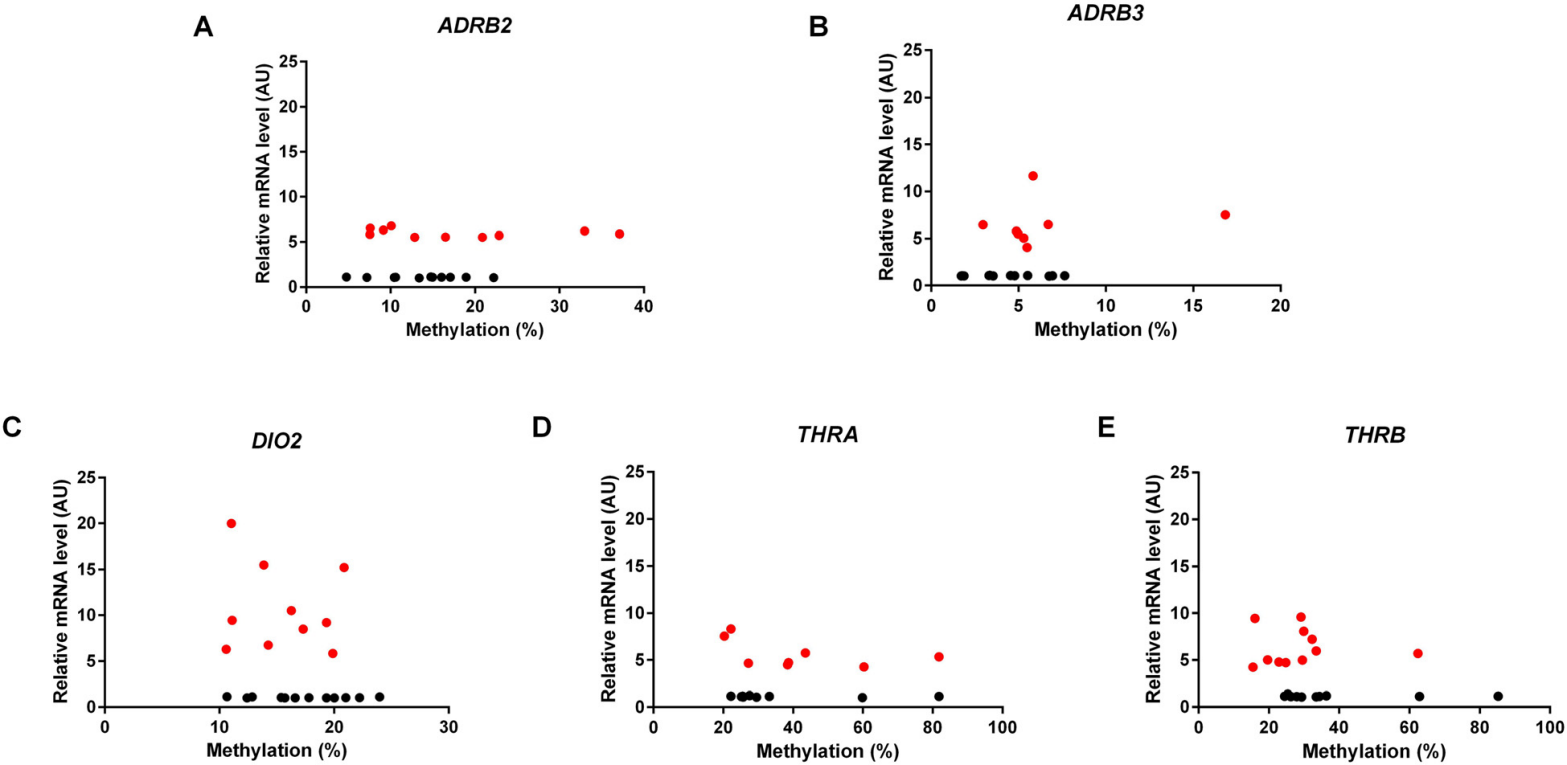
**Correlation of the expression of** ***ADRB2*** **(A),** ***ADRB3*** **(B)** ***, DIO2*** **(C),** ***THRA*** **(D) and** ***THRB*** **(E) with the methylation status of their promoters.** Red dots represent samples with high whereas black – with low expression of the investigated gene.

The analysis of the methylation status of genes of interest was preceded by an *in silico* analysis of their regulatory regions with the CpG Islands Searcher (www.cpgislands.com) and CpG Plot (http://www.ebi.ac.uk). In five out of six genes with expression levels significantly different in obese and slim individuals, namely in *ADRB2*, *ADRB3*, *THRA*, *THRB*, and *DIO2*, the analysis revealed the presence of CpG islands in their promoters. Subsequently, 22 DNA samples for each gene, 11 representing tissues with a low, and 11 representing tissues with a high expression of this gene were used for methylation analysis. The mean level of methylation in tissues with a low expression was similar to the level of methylation in tissues with a high expression of each of these genes. There was no correlation between the levels of expression and methylation (Figure 4). In addition, the mean level of methylation of each gene was similar in VAT and SAT. Finally, the mean levels of methylation of the genes of interest were similar in obese and slim patients; however, the number of tissues from slim patients was only 5 and the statistical power of this analysis was low.

## Discussion

In this work we show that the mean expression of several thermogenesis-related genes is significantly lower in adipose tissues originating from obese patients than in tissues from lean individuals. We also suggest that this phenomenon is probably not associated with differential methylation of their promoters.

The genes investigated in the present study formed three groups. The first consisted of genes encoding β-adrenergic receptors. Both ADRB2 and ADRB3 play an important role in the regulation of lipolysis in human BAT and WAT that provides free fatty acids for thermogenesis. There is indirect evidence that ADRB2 and ADRB3 might participate in the regulation of body weight in humans. For example, their established polymorphisms, e.g. Glu27 variant of *ADRB2* or 64Arg variant of *ADRB3*, both characterized by a reduced receptor activity were associated with metabolic complications and with weight gain in different populations [23-26]. Individuals with a low ADRB3 function in adipose tissue (assessed by its lipolytic activity) tend to increase their body weight over time, whereas a high receptor function seems to protect from weight gain [27]. These data, together with the results from animal studies showing that mice with betaadrenergic receptors knock-outs have reduced metabolic rate and developed massive obesity due to a failure of diet-induced thermogenesis [15] suggest that an unaffected function of ADRB2 and ADRB3 is crucial for thermogenesis and lipolysis in adipose tissue. However, we found that the expression of *ADRB2* and *ADRB3* encoding these receptors was significantly decreased in adipose tissues of obese subjects. We hypothesize that this phenomenon may contribute to the development of obesity and speculate that this might result in a lower effectiveness of receptor-stimulating compounds in the treatment of this condition. Notably, we did not observe differences in the *ADRB1* expression between obese and normal-weight study participants. This finding is consistent with previous data showing no association of polymorphic variants of this gene with body mass in humans, and with the fact that the blockage of ADRB1 did not inhibit cold-induced thermogenesis in humans [28].

The second analyzed group consisted of genes encoding proteins involved in the metabolism (DIOs) and function (TRs) of thyroid hormones, the most important hormones involved in energy production in mitochondria. Studies in animals have demonstrated that knockout of *dio2* increased the susceptibility to diet-induced obesity while animals with *thra* knockout were highly vulnerable to low temperatures due to the complete lack of BAT reactivity to noradrenergic stimulation [14,16]. Genetic studies performed in humans suggested that functional polymorphisms of *DIO2* and *THRA* that decrease gene expression and/or protein activity might increase susceptibility to obesity and its complications [29,30]. Low expression of *DIO2*, *THRA*, and of *THRB* in adipose tissues of obese individuals might then be a mechanism related to the progression of obesity, difficulties in weight loss and, supposedly, resistance to the therapies targeting this pathway [31]. This hypothesis is consistent with the finding that obese, clinically euthyroid patients commonly have elevated serum levels of free T3 (FT3) and decreased expression of *THRA* in adipose tissues compared to lean individuals [32]. One can therefore speculate that the reduced expression of *THRA* and *THRB* in adipose tissue of obese individuals changes the phenotype of adipocyte in such way that it becomes partially “resistant” to T3. A decreased expression of *DIO2* resulting in a lower local conversion of T4 to T3 and a decreased intra-cellular concentration of T3 might also contribute to this phenomenon. As a consequence, the level of FT3 in serum increases to cope with the peripheral hormone “resistance”, but this might be insufficient to normalize the disturbed T3-dependent metabolism in the adipocyte. Notably, we had expected that *DIO1*, which is regulated by TRs, would be also under-expressed in the obese, but our study did not confirm these expectations. Moreover, Ortega *et al.* found that the expression and activity of DIO1 are increased in adipose tissues of obese individuals of Spanish origin [33], suggesting a minor role for T3 in the regulation of *DIO1* expression in adipose tissues of obese individuals.

The third group of analyzed genes was composed of genes encoding uncoupling proteins. Since the expression of all of them is activated by T3 [11,34,35], one could expect that in adipose tissues of obese subjects, where the expression of *THRA*, *THRB*, and of *DIO2* is decreased, all *UCP* genes would be under-expressed. We found however that only the expression of *UCP2* was decreased. Our data corroborate previous findings in humans showing that obesity, low rates of energy expenditure and metabolic complications are linked to certain *UCP2* polymorphisms, such as the presence of 3′UTR *Ins* variant altering mRNA processing or stability [36,37]. In turn, an unchanged expression of *UCP1* and *UCP3* suggests that T3 might not be the most important molecule controlling the activity of these genes in adipose tissues of obese subjects. In fact, other, T3-independent mechanisms may play a dominant role. Indeed, some researchers suggest that a chief regulator of *UCP1* in adipose tissue is PPARγ coactivator-1α [38]. In skeletal muscles, the *UCP3* expression is up-regulated when long-chain fatty acids delivery exceeds their oxidation capacity and MyoD, PPARα and PPARδ transcription factors co-regulate this process; it has not been tested however if similar mechanisms regulate the *UCP3* expression in adipocytes [39].

To date little is known about the differences in thermogenic activity between VAT and SAT. The finding that “beige” adipocytes are present predominantly in human SAT may suggest its dominant role in thermogenesis [40] and support the epidemiological and experimental data indicating that increased subcutaneous fat exerts a lower risk of metabolic complications than visceral fat reviewed in [41]. Our finding that the expression of *DIO2, ADRB2* and *ADRB3* in obese subjects was significantly lower in VAT than in SAT might indicate a lower lipolytic and thermogenic potential of VAT, corroborating previous studies.

Out of many mechanisms regulating gene expression in adipose tissue, epigenetic modifications are of special interest. Since diet is the chief factor influencing the maturation and metabolism of adipocytes, as well as epigenetic modifications, especially methylation of DNA [20-22,42], we decided to investigate if the changes regarding the expression of thermogenesis-related genes in diet-induced obesity can be related to the methylation status of their regulatory regions [43]. However, we found no differences in methylation between normal-weight and obese subjects, between VAT and SAT, and between tissues with high *vs.* low expression of a given gene. In addition, the methylation level of the investigated genes was not related to the level of their expression. This may be due to a number of phenomena. Firstly, methylation may not be the chief mechanism involved in the regulation of activity of these genes in adipose tissue. Secondly, the analyzed regions, although carefully chosen, may not be crucial for the regulation of gene activity. Thirdly, we have analyzed only the overall methylation of the promoter fragments, while the differences in methylation of a specific cytosines located within the binding sites for strong transcriptional activators could also be relevant. Therefore, further studies using other analytical methods are needed to clarify whether or not this epigenetic modification is involved in the regulation of the activity of thermogenesis-related genes in adipose tissue in obesity. In addition, newest data indicate, that drugs often taken by obese individuals can affect epigenetic modifications. For example, it has been demonstrated that metformin might increase [44], while statins might decrease methylation of DNA [45]; this should also be taken into account during data analysis.

## Conclusions

To the best of our knowledge, the evidence we present here is the first to show that adipose tissues of obese individuals are characterized by a decreased expression of the key genes involved in the activation of thermogenesis. On that basis we propose that adipose tissue of obese individuals might be less reactive to both hormonal and adrenergic stimuli and, therefore, less likely to activate thermogenesis than in lean subjects.

## Additional files

Additional file 1: Table S1. Primers used for the analysis of expression of thermogenesis-related genes. Additional file 2: Table S2. Primers used for the analysis of methylation status. Additional file 3: Figure S1. Comparison of the expression of *PPARG* gene in the visceral (VAT) and subcutaneous (SAT) adipose tissues of obese (O) and normal-weight (N) individuals. Results, normalized against the expression of *ACTB*, are shown as the mean ± standard deviation.

## Abbreviations

*ACTB*: Gene encoding β-actin
ADRB: Adrenergic receptor β
*ADRB1*: Gene encoding adrenergic receptor β1
*ADRB2*: Gene encoding adrenergic receptor β2
*ADRB3*: Gene encoding adrenergic receptor β3
AU: Arbitrary units
BAT: Brown adipose tissue
BMI: Body mass index
DIO1: Type 1 5’-iodothyronine deiodinase
*DIO1*: Gene encoding type 1 5’-iodothyronine deiodinase
DIO2: Type 2 5’-iodothyronine deiodinase
*DIO2*: Gene encoding type 2 5’-iodothyronine deiodinase
DM: Diabetes mellitus
FT3: Free triiodothyronine
FT4: Free thyroxin
GFR: Glomerular filtration rate
HDL: High density lipoproteins
IGT: Impaired glucose tolerance
LDL: Low density lipoproteins
PPARα: Peroxisome proliferator-activated receptor alpha
PPARδ: Peroxisome proliferator-activated receptor delta
PPARγ: Peroxisome proliferator-activated receptor gamma
*PPARG*: Gene encoding peroxisome proliferator-activated receptor gamma
SAT: Subcutaneous adipose tissue
TR: Thyroid hormone receptor
TRα: Thyroid hormone receptor α
TRβ: Thyroid hormone receptor β
TREs: Thyroid hormone-response elements
*TRHA*: Gene encoding thyroid hormone receptor α
*TRHB*: Gene encoding thyroid hormone receptor β
TSH: Thyroid stimulating hormone
T3: Triiodothyronine
T4: Thyroxin
UCP: Uncoupling protein
*UCP1*: Gene encoding uncoupling protein 1
*UCP2*: Gene encoding uncoupling protein 2
*UCP3*: Gene encoding uncoupling protein 3
VAT: Visceral adipose tissue
WAT: White adipose tissue
