# Supplementary material for: Obesity is associated with a decrease in expression but not with the hypermethylation of thermogenesis-related genes in adipose tissues
Source: J Transl Med. 2015 Jan 27;13:31. doi: 10.1186/s12967-015-0395-2 (PMC4314800; doi:10.1186/s12967-015-0395-2)
Supplement: Additional file 1: Table S1. — Primers used for the analysis of expression of thermogenesis-related genes. [file 12967_2015_395_MOESM1_ESM.docx]

Additional file 1: Table S1. Primers used for the analysis of expression of thermogenesis-related genes.

| **Gene** | **Primers** | | **Annealing**  **Temperature** |
| --- | --- | --- | --- |
| *ADRB1* | F | 5’ACGCTCCACCAACCTCTTCAT3’ | 61^o^C |
|  | R | 5’CAATGACACACAGGGTCTCG3’ |  |
| *ADRB2* | F | 5’AGGCCTTACCTCCTTCTTGC3’ | 61^o^C |
|  | R | 5’GATCACCAGGGGAACGTAGA3’ |  |
| *ADRB3* | F | 5′ATGGGCACCTTCACTCTCTG3’ | 62^o^C |
|  | R | 5’TGAAGGCAGAATTGGCATAA3’ |  |
| *DIO1* | F | 5’CCTGTGTCCCTAGCTGAATC 3’ | 61^o^C |
|  | R | 5’GTGCCTGTAGAGTGCAACTG 3’ |  |
| *DIO2* | F | 5’TGCTGACCTCAGAGGACTG3’ | 61^o^C |
|  | R | 5’CTGTTTGTAGGCATCGAGGAG3’ |  |
| *THRA* | F | 5’CAAGGCAACTGGTTATCACTAC3’ | 62^o^C |
|  | R | 5’GATTGTGCGGCGAAAGAAG3’ |  |
| *THRB* | F | 5’CCCTTTGTTCTTGGAAGTGTTC3’ | 62^o^C |
|  | R | 5’AGAGCTAGGCAATGGAATGAAA3’ |  |
| *UCP1* | F | 5’TCCCCCGGTGGATGTAGTAAA3’ | 58^o^C |
|  | R | 5’CTTGAAGAAAGCCGTTGGTC3’ |  |
| *UCP2* | F | 5’GAGGTGGTCGGAGATACCAA3’ | 61^o^C |
|  | R | 5’GAGCAACATTGGGAGAGGTC3’ |  |
| *UCP3* | F | 5’CCTGTTTTGCTGACCTCGTT3’ | 62^o^C |
|  | R | 5’ATGGAGGCGAAGCTCATCT3’ |  |
| *PPARG* | F | 5’GGCTTCATGACAAGGGAGTT3’ | 59 ^o^C |
|  | R | 5’AACTCAAACTTGGGCTCCATAAAG3’ |  |
| *ACTB* | F | 5’CAGCCTGGATAGCAACG­TACA3’ | 61^o^C |
|  | R | 5’TTCTACAATGAGCTGCGTGTG3’ |  |

F: forward primer, R: reverse primer.
