# Supplementary material for: Obesity is associated with a decrease in expression but not with the hypermethylation of thermogenesis-related genes in adipose tissues
Source: J Transl Med. 2015 Jan 27;13:31. doi: 10.1186/s12967-015-0395-2 (PMC4314800; doi:10.1186/s12967-015-0395-2)
Supplement: Additional file 2: Table S2. — Primers used for the analysis of methylation status. [file 12967_2015_395_MOESM2_ESM.docx]

Additional file 2: Table **S**2**.** Primers used for the analysis of methylation status.

| **Gene** | **Primers** | | **Annealing**  **Temperature** |
| --- | --- | --- | --- |
| *ADRB2* | F | 5’ CACCACACCCACACCACA3’ | 64^o^C |
|  | R | 5’ATTGGGTGCCAGCAAGAAG3’ |  |
| *ADRB3* | F | 5′CCTTCCTTCTTTCCCTACCG3’ | 64^o^C |
|  | R | 5’TGGTCTGGAGTCTCGGAGTC3’ |  |
| *DIO2* | F | 5’GACTCGGTCATTCTGCTCAAG3’ | 62^o^C |
|  | R | 5’CTGTTTGTAGGCATCGAGGA 3’ |  |
| *THRA* | F | 5’GCTCACTCGCACTCACACC3’ | 62^o^C |
|  | R | 5’CTCGCTCCCTCCTCCTTAC3’ |  |
| *THRB* | F | 5’AAGTCGGACAGCCGTGAG3’ | 64^o^C |
|  | R | 5’CTGGGGCACCAGAGTCC3’ |  |

F: forward primer, R: reverse primer
