# Supplementary figures and images for: Obesity is associated with a decrease in expression but not with the hypermethylation of thermogenesis-related genes in adipose tissues
Source: J Transl Med. 2015 Jan 27;13:31. doi: 10.1186/s12967-015-0395-2 (PMC4314800; doi:10.1186/s12967-015-0395-2)

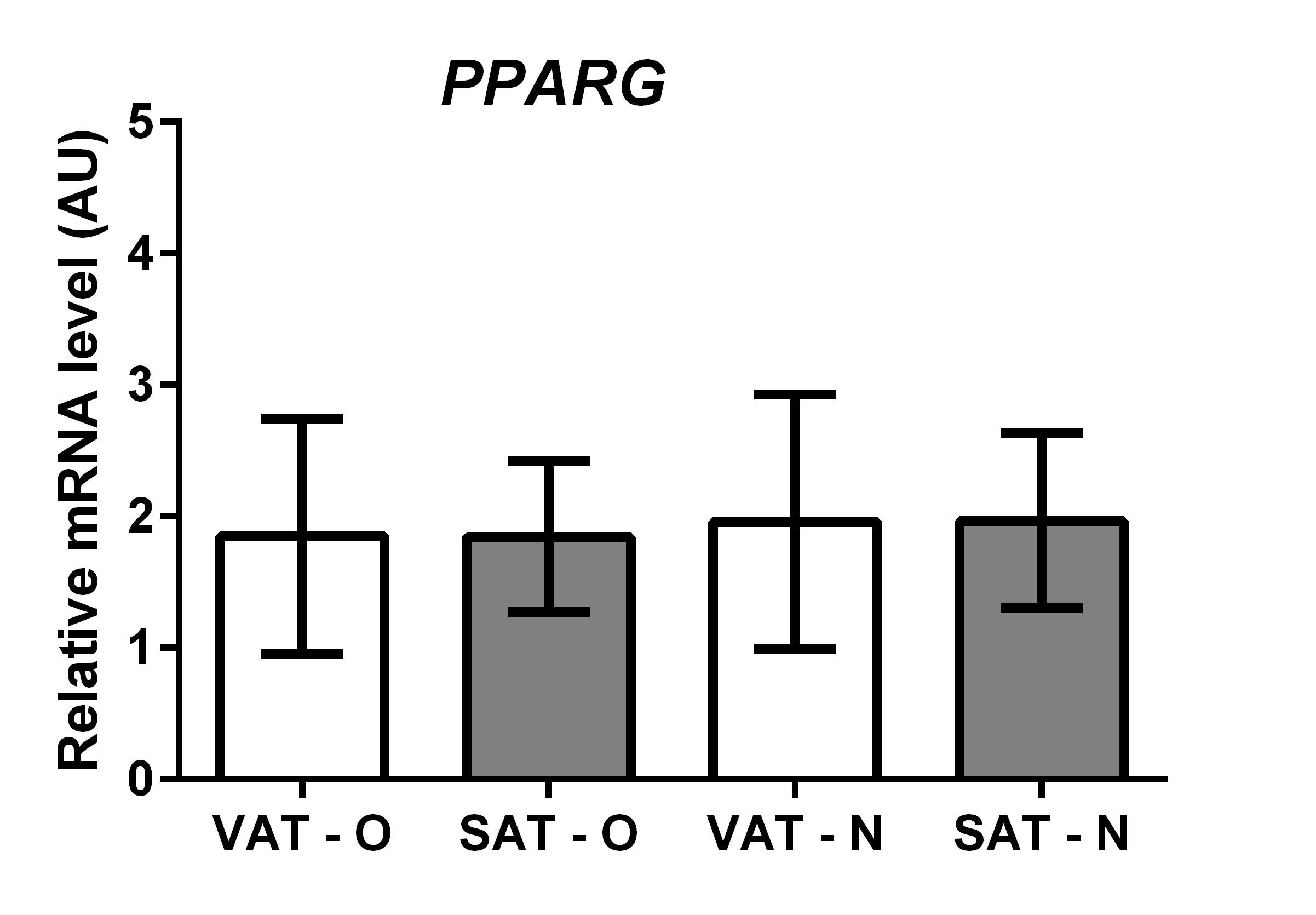

Supplement: Additional file 3: Figure S1. — Comparison of the expression of PPARG gene in the visceral (VAT) and subcutaneous (SAT) adipose tissues of obese (O) and normal-weight (N) individuals. Results, normalized against the expression of ACTB, are shown as the mean ± standard deviation. [file 12967_2015_395_MOESM3_ESM.jpeg]
